# Metabolomic Signatures of Commercial Ready-to-Drink Beverages by Dual-Mode Untargeted LC–MS/MS

**DOI:** 10.3390/metabo16060404

**Published:** 2026-06-10

**Authors:** Ivana Blaženović, Kara Bresnahan, Shunyang Wang

**Affiliations:** 1 Adikai Insights, San Mateo, CA 94403, USA; 2 Kara Bresnahan Regulatory and Scientific Affairs Consulting, Madison, WI 53701, USA; bresnahan.kara@gmail.com; 3 Department of Chemistry, University of California Davis, Davis, CA 95616, USA; sywang@ucdavis.edu

**Keywords:** untargeted metabolomics, LC–MS/MS, bioactive compounds, ready-to-drink beverages, functional beverages, polyphenols, molecular profiling

## Abstract

**Background:** The rapid expansion of functional ready-to-drink (RTD) beverages—formulated with prebiotic fibers, botanical extracts, and reduced sugar—has outpaced systematic characterization of their small-molecule composition. **Methods:** We applied dual-mode untargeted high-resolution liquid chromatography–tandem mass spectrometry (LC–MS/MS), integrating hydrophilic interaction (HILIC) and reversed-phase C18 separations, to profile five commercial RTD beverages spanning distinct formulation categories: Coca-Cola^®^, Poppi^®^ Orange, OLIPOP^®^ Cream Soda, Pure Leaf^®^ Unsweetened Black Tea, and BeePop™ Peach + Orange Blossom Honey. **Results:** Across all products, 478 compounds were structurally annotated at Metabolomics Standards Initiative (MSI) Levels 1 and 2, of which 42 matched compounds with reported bioactivity in a curated literature-based reference database. Seventeen compounds—including the NAD+ precursor trigonelline and multiple B vitamins—were detected across all five products. The number and diversity of compounds with reported bioactivity varied substantially by product and correlated with botanical ingredient complexity. **Conclusions:** This work presents a qualitative molecular survey of the RTD beverage category using standardized, dual-mode untargeted metabolomics, providing a reference dataset for future targeted quantitation studies.

## 1. Introduction

Global sales of ready to drink (RTD) beverages exceeded USD 500 billion in 2023 and continue to grow, driven in part by consumer demand for products perceived as healthier alternatives to traditional carbonated soft drinks [1]. In response, a new wave of functional sodas—typified by brands such as Poppi^®^ and OLIPOP^®^—has entered the mainstream market, promising digestive support, reduced glycemic impact, and prebiotic benefits. At the same time, botanical and naturally derived beverages (e.g., honey, green tea, and fermentation-derived formulations) occupy adjacent shelf space with claims anchored to plant bioactives.

These marketing narratives raise a fundamental scientific question: Do the compositional differences among RTD beverage categories translate into measurable differences in bioactive compound content? Labeling reveals ingredients intentionally added to RTD beverages and may highlight key small molecules, but does not reflect the full breadth of the low-molecular-weight chemistry present in a finished beverage—including endogenous plant metabolites, fermentation by-products, Maillard reaction products, and intact phytochemicals from botanical inputs [2,3]. Answering this question requires a technology that is agnostic to prior hypotheses: untargeted metabolomics.

High-resolution untargeted LC–MS/MS, when combined with comprehensive spectral libraries and in silico annotation tools, enables the detection and putative identification of hundreds to thousands of distinct small molecules in a single sample [2,4,5]. Community-defined confidence tiers from the Metabolomics Standards Initiative (MSI) provide a framework for communicating structural annotation certainty, distinguishing confirmed identifications (Level 1, matched to authentic standards) from high-confidence spectral matches (Level 2) and class-level putative annotations (Level 3) [3,6]. Restricting bioactive interpretations to MSI Levels 1–2 preserves structural rigor and avoids overclaiming from the large fraction of the detected metabolome that remains unresolved—what has been termed the ‘dark metabolome’ [3,6].

Prior work has characterized specific compound classes in individual beverage types—catechins and theaflavins in tea [7,8], polyphenols in coffee [9], organic acids in fermented beverages [10], and bioactives in almonds [11]—but no study has applied a unified, dual-mode LC–MS/MS workflow across beverages spanning legacy carbonated soft drinks (CSDs), prebiotic sodas, and botanical-derived drinks within a single analytical framework.

Many beverage-derived compounds fall within phytochemical classes such as polyphenols and related plant bioactives. Bioactives have been defined by the United States Department of Health and Human Services as “constituents in foods and dietary supplements, other than those needed to meet basic human nutritional needs, that are responsible for changes in health status” [12]. Their biological relevance depends on dose, metabolism, and bioavailability, and detection alone does not indicate health benefits. Accordingly, a composition-first analytical framework avoids binary or evaluative interpretations. Throughout this manuscript, “bioactive” refers to compounds for which biological activity has been reported in peer-reviewed literature; detection in a beverage matrix does not imply that the compound is bioavailable within the product matrix nor present at a biologically efficacious concentration.

This research addresses three primary objectives: (i) to construct an untargeted molecular profile of five commercially available RTD beverages representing major modern product categories using dual-mode LC–MS/MS; (ii) to systematically map detected metabolites against a curated bioactive reference database anchored in clinical and mechanistic evidence (Supplementary Table S2); and (iii) to characterize and compare bioactive diversity—in terms of compound identity and biological annotation—across these formulation types. The resulting dataset provides a foundation for nutrition science and functional ingredient discovery and identifies candidate compounds for future targeted validation and quantification.

## 2. Materials and Methods

### 2.1. Beverage Selection and Sample Preparation

Five commercially available RTD beverages were selected to span the major formulation paradigms in the current US RTD market: Coca-Cola^®^ (produced in USA, a legacy caramel-colored CSD) as a conventional baseline; Pure Leaf^®^ Tea (produced in USA, unsweetened black tea flavor, no added sweetener nor botanical additives beyond the tea) as a minimally processed botanical reference; Poppi^®^ (produced in USA, orange flavor, a prebiotic soda containing apple cider vinegar and inulin) and OLIPOP^®^ (produced in USA, cream soda flavor, a prebiotic soda with botanical extracts and plant fiber) as two prebiotic functional sodas employing distinct ingredient strategies; and BeePop™ (produced in USA, peach + orange blossom honey flavor, a carbonated fermented green-tea derived beverage with prebiotic fiber that is sweetened with juice and honey) representing an emerging category of fermentation-derived soda alternatives. The selection is illustrative, not exhaustive, and results should not be generalized beyond these specific products. Four analytical replicates (*n* = 4) were prepared from a single container of each beverage (i.e., from the same production lot). These technical replicates assess analytical reproducibility but do not capture batch-to-batch, seasonal, or manufacturer-level variability. All products except BeePop™ were purchased from a single US retail location, and BeePop™ was shipped for analysis by the manufacturer. Commercial beverages are manufactured under standardized formulations and quality-control protocols designed to ensure batch-to-batch consistency; the profiles reported here are therefore expected to be representative of the products as marketed, though confirmation across multiple production lots would strengthen this assumption. All samples were handled under standardized laboratory conditions to minimize contamination and carryover.

For sample preparation, each beverage was diluted 1:1 (*v*/*v*) with LC–MS-grade acetonitrile, vortex-mixed for 30 s, centrifuged at 13,000× *g* for 5 min, and the clarified supernatant was transferred to polypropylene LC–MS vials. This monophasic extraction strategy is established for broad small-molecule coverage across polarity ranges while reducing matrix-related signal suppression [2,13,14].

### 2.2. LC–MS/MS Data Acquisition

Untargeted LC–MS/MS data were acquired using two orthogonal chromatographic modes on a Thermo Scientific Exploris platform with heated electrospray ionization (HESI): (i) HILIC separation (Waters ACQUITY Premier UPLC BEH Amide, 50 × 2.1 mm, 1.7 µm) for polar and highly water-soluble metabolites, and (ii) reversed-phase C18 separation (Acquity Premier BEH C18 1.7 µm VanGuard FIT, 2.1 × 50 mm) for less polar and lipid-like molecules. Both positive and negative ionization modes were acquired. HESI parameters: spray voltage 2.5 kV (positive)/3.5 kV (negative); capillary temperature 290 °C; sheath gas 60 au; probe temperature 475 °C.

Data-dependent acquisition (DDA) MS/MS was performed using a normalized collision energy of 25 eV and a 1.4 Da isolation window. MS/MS spectra were acquired at 120,000 (for MS1) and 60,000 (for MS2) resolutions. Iterative inclusion/exclusion lists were applied using AcquireX (Thermo Fisher Scientific, Waltham, MA, USA) across five successive injections per platform to maximize unique MS/MS coverage. Background ions were characterized via solvent-blank and extraction-blank injections and excluded from subsequent acquisitions.

Analytical quality was ensured through deuterated internal standards spiked prior to extraction, pooled quality-control (QC) samples injected at regular intervals to monitor instrument drift, randomized injection order, and mass accuracy calibration of the Orbitrap analyzer before each acquisition batch, following established protocols [14].

### 2.3. Data Processing and Annotation

Peak detection, alignment, and annotation were performed in MS-DIAL (version 5.2) [4]; molecular formula assignment applied seven-rule heuristic filtering [15]. Processing parameters: MS1 tolerance 0.01 Da; MS2 tolerance 0.025 Da; minimum peak height 10,000 counts; smoothing method linear weighted moving average (level 2); MS/MS identification score cutoff ≥80% with ≥3 fragment ions matching a reference spectrum; alignment retention-time tolerance 0.05 min; alignment MS1 tolerance 0.01 Da. Feature tables were exported and subjected to blank-subtraction (sample/blank peak height ratio ≥ 10) and replicate-variability filtering (RSD ≤ 40% across all four replicates per beverage). All inter-beverage comparisons reported herein are qualitative (presence/absence after replicate-consistency filtering) or at most semi-quantitative. Peak intensities are reported in arbitrary units and are not calibrated against external standards; they should not be interpreted as concentrations. Because beverage matrices differ in pH, ionic strength, sugar content, and co-extracted compounds, differential ion suppression or enhancement may affect detection sensitivity across products. The absence of a compound in a given beverage therefore indicates non-detection under these analytical conditions, not confirmed absence.

Spectral library matching incorporated HMDB, NIST23, MoNA, and MassBank reference collections [16,17,18]. In silico structure prediction used SIRIUS (version 6.1.0.) [19]; chemical taxonomy was assigned with ClassyFire [20]. Annotation confidence was reported per MSI guidelines: Level 1 (confirmed with authentic reference standards), Level 2 (MS/MS spectral match against library), and Level 3 (compound class predicted by in silico tools). For all bioactive analyses and comparative interpretations, only MSI Level 1–2 annotations were used.

### 2.4. Bioactive Reference Database and Mapping

An internal bioactive reference database was developed by compiling small molecules with published evidence of biological activity from peer-reviewed literature (PubMed) and publicly registered clinical trial records (ClinicalTrials.gov, accessed 20 January 2026). Inclusion criteria: a compound was entered into the database if (a) it had a confirmed chemical structure with a registered InChIKey, (b) at least one peer-reviewed publication reported a biological effect in a model system (in vitro, animal, or human), and (c) human pharmacokinetic data demonstrating systemic bioavailability were available. For each compound, the database captures biological benefit category (e.g., antioxidant, hypoglycemic, neuroprotective), primary endpoints from key studies, study design and population, evidence strength classification, and effect direction. Evidence strength was classified as confirmatory if ≥2 independent human RCTs or a published meta-analysis reporting a statistically significant effect were identified; suggestive if ≥1 human interventional or observational study with a relevant endpoint was identified; and preclinical, where evidence was limited to in vitro or animal models with no published human efficacy data. These classifications involve judgment and should be considered approximate rather than definitive. Chemical identifiers were standardized using InChIKey strings; database matching used the first InChIKey block (molecular connectivity layer) to ensure structurally equivalent hits across ionization states and stereochemical variants. Detected MSI Level 1–2 features were mapped against this database after blank subtraction and replicate-consistency filtering. Additionally, for molecules not captured in the database but detected in the beverages, PubMed was searched for human clinical trials, meta-analyses, and pharmacokinetic studies (search terms: compound name + “human” + “randomized controlled trial” OR “meta-analysis” OR “clinical trial”). Evidence strength was graded using the existing framework based on study design quality and reproducibility of reported effects. Only compounds with at least preclinical evidence and confirmed human pharmacokinetic data (demonstrating systemic bioavailability) were added. Five compounds achieved Suggestive-level evidence (trigonelline [21], theobromine [22], ferulic acid [23], naringin [24], and resveratrol [25]) and three achieved Preclinical-level evidence (myricetin, gallic acid, and protocatechuic acid) after this screening. The complete database (204 compounds) is provided as Supplementary Table S2, including all source references, to enable independent evaluation and replication. Presence in the bioactive database indicates that a compound has reported biological associations in the literature; it does not imply that beverage-relevant concentrations are biologically efficacious. Quantitative dose–response assessment and bioavailability studies were beyond the scope of the current study and should be performed separately.

### 2.5. Chemical Diversity Analysis

Chemical classification was performed using ClassyFire, a freely available tool that allows classification of molecules on several levels [20]. Shannon diversity index (H) was computed at the superclass level as H = −Σ p_i_ ln(p_i_), where p_i_ is the proportion of molecules assigned to superclass i. Molecule counts used in diversity calculations applied blank subtraction, replicate-consistency, and CV ≤ 40% filters. Principal component analysis (PCA) was performed on log10-transformed, z-score-normalized feature intensities using all 3328 aligned features (*n* = 4 replicates per beverage).

## 3. Results

### 3.1. Global Molecular Coverage

Dual-mode LC–MS/MS analysis of five RTD beverages yielded approximately 3328 aligned features across all samples prior to structural annotation. Of these, 478 were structurally annotated at MSI Level 1 (*n* = 347, confirmed with authentic standards) or Level 2 (*n* = 131, spectral library match) and constitute the analytical dataset reported here. The annotated dataset of 478 features (L1 + L2) is the basis for all compound-level comparisons reported herein; per-beverage counts with confidence-level breakdown are presented in Section 3.2. In the full pre-annotation detection run, 1457 of ~3328 total features were detected across all five beverages, indicating a substantial shared chemical core irrespective of formulation category.

**Figure 1:**
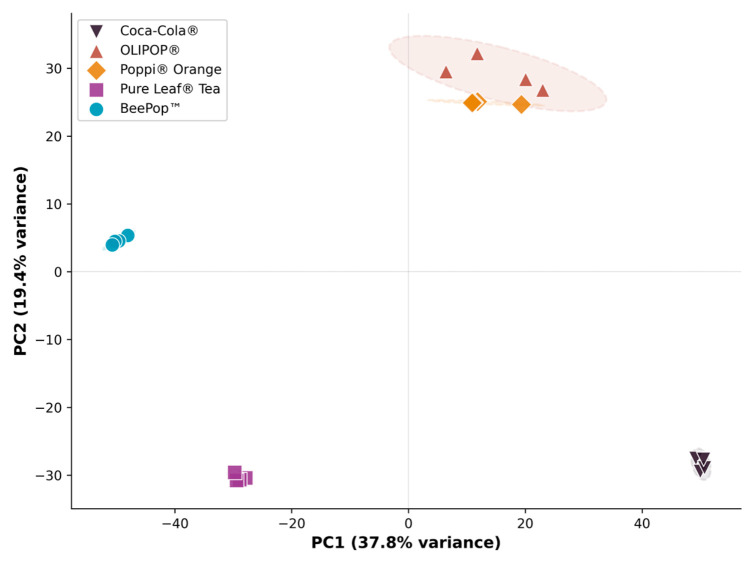
Principal Component Analysis of RTD Beverage Metabolomes. PCA score plot of all five RTD beverages (*n* = 4 replicates per beverage) computed on log_10_-transformed, z-score normalized intensities of all ∼3328 detected features (pre-annotation). PC1 explains 37.8% of variance; PC2 explains 19.4% (cumulative 57.2%). Ellipses represent 95% confidence intervals per beverage group. Tight replicate clustering confirms high analytical reproducibility; clear inter-group separation confirms genuine compositional divergence across formulation categories.

PCA performed on all ~3328 detected features prior to annotation filtering (Principle Component (PC) 1: 37.8% variance; PC2: 19.4%; cumulative 57.2%; Figure 1) demonstrated clear cluster separation by beverage type with tight within-group replicate clustering, confirming high analytical reproducibility and genuine inter-beverage compositional divergence. Coca-Cola^®^ separated strongly along the positive PC1 axis; BeePop™ and Pure Leaf^®^ Tea occupied the negative PC1 region; OLIPOP^®^ and Poppi^®^ Orange clustered together in the positive PC1 space but were resolved along PC2.

These patterns indicate that global molecular fingerprints reflect formulation origin—caramel-coloring chemistry in legacy CSDs, tea phytochemistry in brewed beverages, and botanical/fermentation inputs in the functional soda category. The PCA is presented as a descriptive visualization of inter-beverage compositional differences and within-group analytical reproducibility. With *n* = 4 replicates per beverage and ~3328 features, the analysis is underdetermined and should not be interpreted as statistically inferential. The tight within-group clustering supports analytical reproducibility but does not constitute a formal statistical test of inter-beverage differences.

### 3.2. Annotation Confidence Distribution

**Figure 2:**
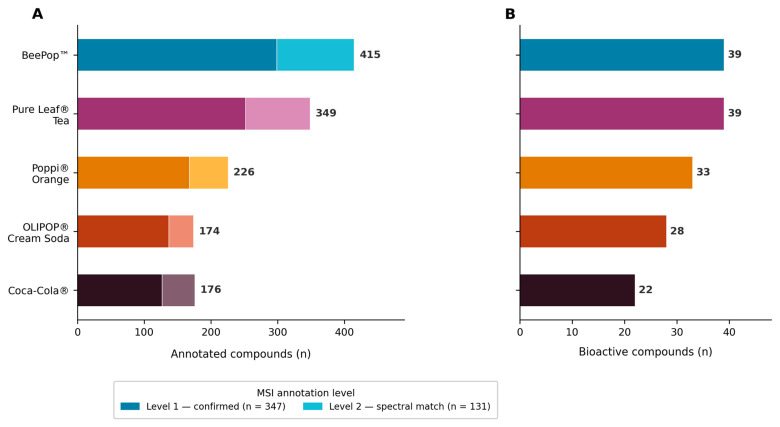
Annotated Molecular Coverage and Bioactive Compound Counts per Beverage. (**A**) Total annotated features (MSI Level 1 + Level 2) per beverage after replicate-presence filtering (≥4/4 replicates, abundance > 10,000), ordered from highest to lowest molecular coverage. Level 2 annotations are putative identifications based on spectral similarity and should be considered tentative. (**B**) Number of bioactive compounds matched to the curated 204-compound bioactive reference database per beverage. BeePop™ and Pure Leaf^®^ Tea tied for the highest bioactive count (39 each). Bars represent absolute compound counts.

The analytical dataset of 478 structurally annotated features comprises MSI Level 1 (L1; confirmed, *n* = 347) and Level 2 (L2; spectral library match, *n* = 131) annotations. After replicate-presence filtering, per-beverage counts are as follows: BeePop™ 415 (L1 = 299, L2 = 116), Pure Leaf^®^ Tea 349 (L1 = 252, L2 = 97), Poppi^®^ Orange 226 (L1 = 168, L2 = 58), OLIPOP^®^ 174 (L1 = 137, L2 = 37), and Coca-Cola^®^ 176 (L1 = 127, L2 = 49). The 478 structurally annotated compounds reported here represent those for which structural identity can be supported at a confidence level appropriate for bioactivity interpretation (Figure 2).

### 3.3. Bioactive Compound Landscape

#### 3.3.1. Overall Bioactive Counts

**Figure 3:**
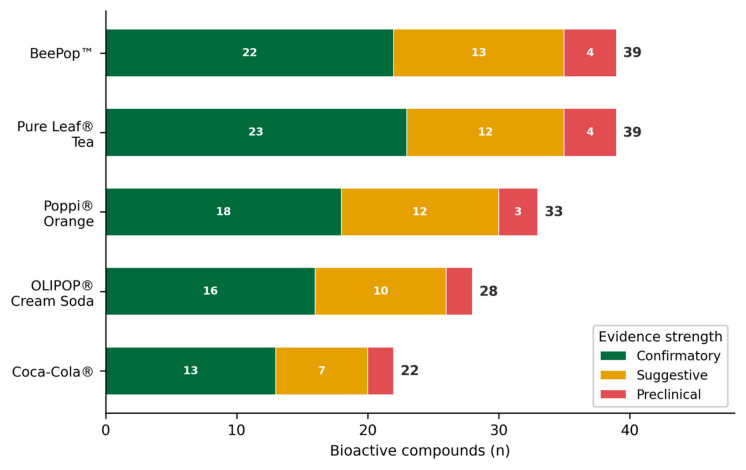
Bioactive Compound Counts and Evidence Strength Distribution. Per-beverage bioactive counts (*n* = 42 total, 8.8% of the 478-compound annotated dataset; 29 at MSI Level 1, 13 at MSI Level 2) stratified by evidence strength classification: Confirmatory (human RCT or meta-analysis with consistent positive-direction results), Suggestive (human observational or mechanistic evidence), and Preclinical (in vitro or animal model evidence only). Level 2 annotations are tentative and require confirmation. All 42 bioactives were detected in the positive evidence direction. Beverages are ordered by total bioactive count.

Mapping MSI Level 1–2 annotated features against a literature-expanded curated bioactive reference database (204 unique compounds) identified 42 compounds with reported bioactivity (8.8% of the 478-compound annotated dataset; 29 at MSI Level 1, 13 at MSI Level 2). Level 2 annotations are putative and may reflect isobaric or isomeric compounds with similar fragmentation patterns; these identifications should be considered tentative until confirmed by targeted analysis. These 42 compounds were detected across all five beverages after applying the replicate-presence filter (Figure 2 and Figure 3; Table 1). Per-beverage counts were as follows: Pure Leaf^®^ Tea 39 = BeePop™ 39 > Poppi^®^ Orange 33 > OLIPOP^®^ 28 > Coca-Cola^®^ 22. Of these 42 bioactives, 17 were robustly present in all five beverages, 39 were shared across at least two beverages, and only 3 were detected in a single beverage, indicating that shared detection of compounds with reported bioactivity—rather than formulation-specific accumulation—was observed across the five beverages tested at current detection thresholds.

#### 3.3.2. The Universal Bioactive Core

Seventeen bioactive compounds were robustly detected in every beverage regardless of formulation category (Table 2), including vitamins B1, B6, and C, amino acid-derived neuroactive precursors, osmolytes, and the plant alkaloid trigonelline. Trigonelline was detected across all five products, including Coca-Cola^®^—a somewhat unexpected finding. This detection is an MSI Level 2 annotation and would benefit from confirmation by targeted analysis with an authentic trigonelline standard. The presence of L-theanine across all five beverages, including OLIPOP^®^ and Coca-Cola^®^, similarly warrants confirmatory targeted analysis.

#### 3.3.3. Beverage-Specific Bioactive Profiles (Figure 4)

Pure Leaf^®^ Tea (39 bioactives) exhibited the richest tea-characteristic phytochemical profile, including catechins (epicatechin, epigallocatechin gallate), theaflavin-related compounds, quercetin, rutoside, silibinin, chlorogenic acid (unique to this beverage), and isoleucine (unique to this beverage). The dominance of oxidatively coupled polyphenols—catechins and theaflavins—is consistent with the black tea processing mechanism and aligns with the established literature on tea bioactives and their antioxidant, cardiometabolic, and neuroprotective associations [7,8,26].

**Figure 4:**
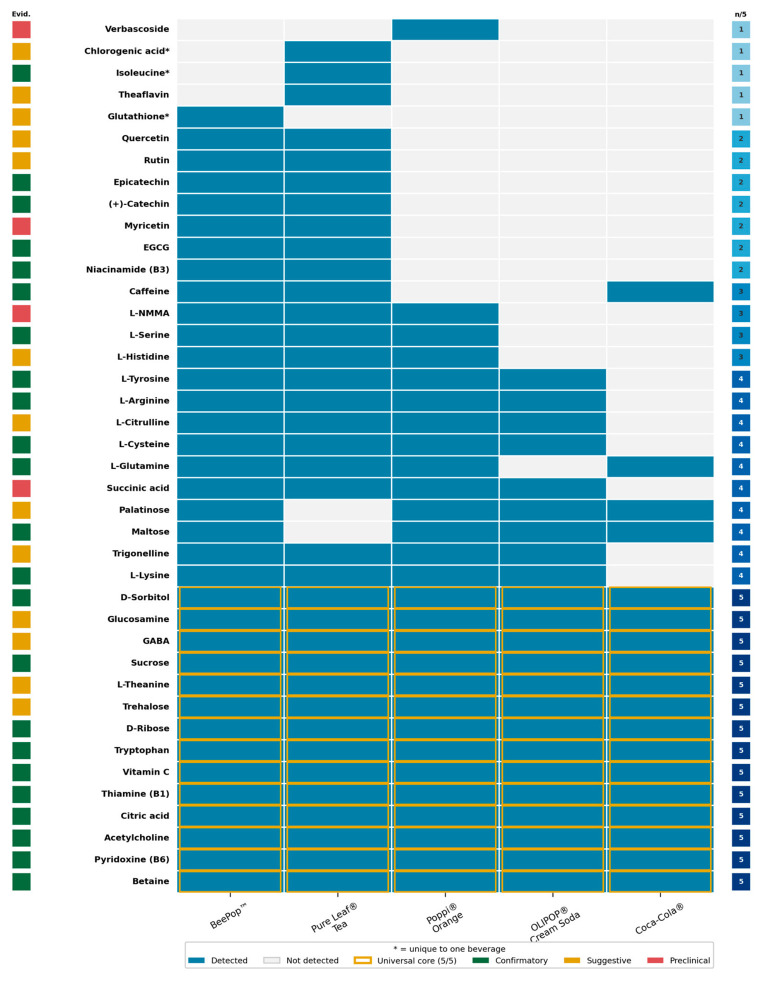
Presence–absence Heatmap of 42 Bioactive Compounds Across Five RTD Beverages. Binary presence (filled) or absence (empty) matrix for all 42 bioactive compounds matched in the curated database after replicate-presence filtering (29 at MSI Level 1, 13 at MSI Level 2, and Level 2 annotations are tentative). Compounds are grouped by chemical superclass. The 17 universally detected compounds (present in all five beverages) are indicated. The three beverage-unique compounds (glutathione, chlorogenic acid, isoleucine) are labeled with asterisks. Beverage columns are ordered by total bioactive count (left to right: BeePop™, Pure Leaf^®^ Tea, Poppi^®^ Orange, OLIPOP^®^, Coca-Cola^®^).

BeePop™ (39 bioactives) displayed the broadest biochemical diversity, with elevated representation across phenolic, flavonoid, amino-acid, and lipid-associated classes. BeePop™ was the sole source of glutathione, a tripeptide with antioxidant properties (unique to this beverage), and showed the highest counts of chlorogenic acid derivatives, luteolin glycosides, and glycosylated phenolic acids. The breadth of BeePop™’s bioactive profile—matching the tea benchmark despite being a carbonated beverage—reflects the multi-botanical ingredient matrix and honey-derived components.

Poppi^®^ Orange (33 bioactives) showed enrichment in fermentation-associated organic acids derived from the apple cider vinegar component—including betaine, verbascoside, and L-NMMA—alongside flavonoids such as epicatechin and quercetin. The organic acid profile is consistent with its apple cider vinegar ingredient declaration.

Coca-Cola^®^ (22 bioactives) contained a substantive bioactive profile including caffeine, citrulline, GABA, choline, L-arginine, histidine, glutamine, N-acetyl-L-leucine, and nicotinamide. Many of these compounds likely derive from the caramel coloring and phosphoric acid processing chemistry, as well as from the natural kola nut extract still present in the formulation. Several of these detections—particularly GABA, citrulline, and L-arginine—are MSI Level 2 annotations and warrant confirmatory targeted analysis. The presence of 22 structurally confirmed bioactive compounds underscores that conventional CSDs are not bioactively inert, though concentrations and dose-response remain to be determined by targeted quantification.

OLIPOP^®^ Cream Soda (28 bioactives) showed a bioactive profile dominated by amino acids, organic acids, and glycan-associated compounds. Prebiotic fiber polymers—OLIPOP^®^ Cream Soda’s primary marketed functional contribution—fall above the small-molecule detection window of this assay and are not captured in the bioactive count.

### 3.4. Beverage-Specific Unique Molecules (Full Feature Space)

Beyond the bioactive subset, unique molecular features in the full annotated feature space (all MSI levels) were as follows: BeePop™ 196 unique molecules > Pure Leaf^®^ Tea 48 > Poppi^®^ Orange 37 > OLIPOP^®^ 34 > Coca-Cola^®^ 28. BeePop™’s 196-feature unique signature—dominated by chlorogenic acid derivatives, luteolin glycosides, and glycosylated phenolic acids at MSI Level 3—represents a pool of currently uncharacterized chemistry that, upon structural elucidation, may yield additional bioactive candidates. This ‘dark metabolome’ contribution from botanical honey-derived sources is qualitatively and quantitatively distinct from the tea, fermentation, or caramel chemistry driving uniqueness in other beverages.

### 3.5. Chemical Superclass Distribution and Diversity

**Figure 5:**
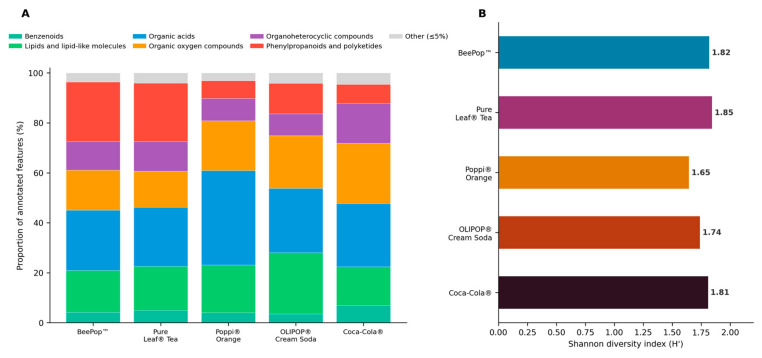
Chemical superclass distribution and Shannon diversity indices. (**A**) Stacked bar chart showing the proportional representation of ClassyFire chemical superclasses across annotated features (MSI Level 1 + 2) per beverage. (**B**) Shannon diversity index (H) calculated at the superclass level for each beverage (H = −Σ p_i_ ln(p_i_)). The compressed range (1.61–1.80) indicates broadly comparable superclass diversity across formulation categories despite substantial differences in ingredient sourcing.

Carboxylic acids and derivatives constituted the most abundant structural superclass across all beverages (Figure 5; Supplementary Figure S1). Shannon diversity indices at the superclass level were narrow (range 1.61–1.80; Figure 5). These indices quantify the evenness of chemical superclass representation among detected compounds and do not reflect functional potency, nutritional value, or biological relevance: Pure Leaf^®^ Tea 1.80 > BeePop™ 1.78 > Coca-Cola^®^ 1.65 > Poppi^®^ 1.64 > OLIPOP^®^ 1.61. The compressed range indicates broadly comparable superclass balance across beverage types despite formulation differences, suggesting that chemical diversity at coarse taxonomic levels is an insufficient discriminator of functional beverage quality. The biologically relevant discriminant is compound identity and evidence depth within shared superclasses, not superclass richness per se.

## 4. Discussion

### 4.1. The Bioactive Hierarchy of RTD Beverages

The most consequential finding of this study is a clear, data-driven bioactive hierarchy among five representative RTD beverages. Botanical and tea-containing formulations (BeePop™, Pure Leaf^®^) yielded the highest number of putatively identified bioactive compounds, consistent with the known phytochemical complexity of botanical plant matrices and the reported bioactivity of tea catechins [7,8]. However, the near-parity between BeePop™—a carbonated beverage—and Pure Leaf^®^—an unflavored brewed tea—challenges the implicit assumption that carbonation or processing necessarily diminishes bioactive content. The botanical and honey-derived ingredient matrix of BeePop™ may contribute a bioactive complement comparable in count to that of unprocessed tea, although this comparison is based on qualitative detection and not on quantified concentrations. Detection counts do not reflect concentrations; targeted quantification is needed to determine whether these qualitative differences translate to differences in biological exposure.

Differences between Poppi^®^ Orange (33 bioactives) and OLIPOP^®^ Cream Soda (28 bioactives) likely reflect differences in ingredient sourcing—particularly the vinegar-derived and phenolic-contributing components of Poppi^®^ versus the fiber-forward formulation strategy of OLIPOP^®^, whose prebiotic activity (inulin, chicory root, nopal cactus) resides in high-molecular-weight polysaccharides not captured by the current small-molecule workflow. These findings underscore the importance of analytical methodology in bioactive evaluation: a polysaccharide-optimized assay would be needed to characterize the high-molecular-weight bioactives not captured by the current workflow.

The detection of 22 structurally confirmed bioactives in Coca-Cola^®^ is a finding that merits careful interpretation. These include compounds with well-established biological associations: caffeine (alertness, focus), citrulline (nitric oxide precursor), GABA (neurotransmitter), choline (nootropic, anti-inflammatory), L-arginine (vasodilator, nitric oxide donor), histidine (hypoglycemic, anti-inflammatory), and nicotinamide (vitamin B3). The most parsimonious explanation for many of these identifications is derivation from the complex chemistry of caramel coloring (Class IV, the most thermally processed), which introduces a heterogeneous mixture of heterocyclic and Maillard reaction compounds that may co-annotate with bioactive structures sharing spectral similarity. Authentic-standard confirmation (MSI Level 1 upgrade) of the most surprising identifications—particularly GABA, citrulline, and L-arginine in a legacy CSD—is warranted, as well as quantification, before functional claims could be considered. Nonetheless, the data indicate that Coca-Cola^®^ contains a diverse repertoire of small molecules, some of which match known bioactive structures at high spectral confidence. Whether these detections reflect genuine bioactive content or spectral co-annotation with structurally similar Maillard reaction products remains to be resolved.

### 4.2. Polyphenols as the Primary Functional Differentiator

Across all bioactive comparisons, phenolic and flavonoid compounds—epicatechin, epigallocatechin gallate (EGCG), quercetin, rutoside, chlorogenic acid, silibinin, beta-cryptoxanthin, and verbascoside—emerged as the primary differentiators between formulation categories. These compounds were concentrated in Pure Leaf^®^ Tea and BeePop™ and are largely absent from OLIPOP^®^ and substantially reduced in Coca-Cola^®^. Catechins such as epicatechin and EGCG fall within the flavan-3-ol class, for which an Expert Panel convened by the Academy of Nutrition and Dietetics concluded that moderate evidence supports cardiometabolic protection at dietary intake levels in the range of 400–600 mg/d [7]. Polyphenol bioactivity is well studied, with evidence supporting roles for epicatechin in cardiometabolic health [7], EGCG in antioxidant and anti-inflammatory pathways [26], and quercetin in vascular and metabolic endpoints [27]. Targeted quantitation studies are required to determine whether polyphenol levels in BeePop™ and Pure Leaf^®^ Tea approach the doses used in the clinical evidence base.

### 4.3. The Universal Core: Shared Biology Across All Formulations

The seventeen universally detected bioactives reveal a shared molecular baseline that persists across all five formulation types tested. Trigonelline warrants focused attention among the universal core. A 2024 Nature Metabolism study by Membrez et al. [21] identified trigonelline as a direct NAD^+^ precursor—the first dietary compound outside the classical niacin/tryptophan pathway shown to raise NAD^+^ in human skeletal muscle. Serum trigonelline levels were inversely correlated with sarcopenia severity, grip strength, and mitochondrial oxidative phosphorylation in a cohort of older adults; supplementation restored muscle function in preclinical aging models. The detection of trigonelline in all five beverages tested here—including Coca-Cola^®^, whose formulation does not include any declared botanical or coffee-derived ingredient—suggests that trigonelline may enter the RTD matrix through caramel coloring precursors, trace coffee-derived flavor compounds, or as a Maillard reaction by-product; this detection is MSI Level 2 and warrants targeted confirmation. L-theanine, tryptophan, and trehalose are compounds with neuroprotective and mood-modulating associations [28,29,30]; their presence across all five products implies that certain bioactive exposures from RTD beverages may be more uniform across categories than previously appreciated, though the functional significance of these contributions cannot be assessed without quantitation. False-positive annotations are an inherent risk in untargeted metabolomics, particularly for MSI Level 2 identifications that rely on spectral similarity without retention-time matching to authentic standards. Structural isomers, in-source fragments, and adduct misassignments can produce high-scoring spectral matches to incorrect library entries. The unexpected detections reported here—L-theanine in non-tea beverages, GABA and citrulline in a legacy CSD, trigonelline across all products—should be regarded as findings warranting targeted confirmation.

### 4.4. Bioactive Exposure in the Context of Macronutrient Profiles

Table 3 displays the macronutrient profile of each beverage as reported in the product’s Nutrition Facts Panel per 12 fluid ounces. In the absence of fat and protein, caloric content is driven entirely by carbohydrates. Pure Leaf^®^ Tea is devoid of calories and carbohydrates. Coca-Cola^®^ contains the highest caloric content (140 kcal), driven exclusively by added sugars (39 g). BeePop™, OLIPOP^®^, and Poppi^®^ contain 64–79% fewer calories than Coca-Cola^®^ due to considerably lower added sugar content; these three beverages also contribute dietary fiber (2–6 g per 12 fl oz). Beyond macronutrient composition, the biological significance of detecting these compounds cannot be assessed without quantitative data. Many of the detected compounds are hydrophilic, rapidly absorbed, and efficiently excreted; their transient pharmacokinetic profiles may preclude meaningful accumulation from a single beverage serving.

Dietary fiber supports digestion, metabolic health, and gut microbiome diversity [13]. It is recognized as a nutrient of public health concern by the Dietary Guidelines for Americans, as more than 90% of US adults do not meet recommended intake levels [31]. BeePop™, OLIPOP^®^, and Poppi^®^ contribute to intakes of this shortfall nutrient in addition to providing exposure to bioactive phenolic plant compounds—a potential dual health benefit not fully reflected in product labeling. This macronutrient context is essential for interpreting the bioactive data: beverages that simultaneously reduce added sugar exposure, contribute fiber, and contain detectable bioactive compounds may represent a compositionally differentiated profile relative to legacy carbonated soft drink (CSD) formulations, though the biological significance of the detected bioactives remains to be established through quantitative analysis.

### 4.5. Implications for Functional Beverage Development and Ingredient Strategy

From an ingredient discovery and product development perspective, each beverage in this study tells a distinct molecular story shaped by its unique ingredient sourcing and formulation philosophy. BeePop™’s 196-feature unique molecular profile reflects its botanical and honey-based inputs and constitutes an uncharacterized chemical space where novel bioactive compounds may reside. Systematic dereplication of this pool through authentic reference standards, preparative isolation, and NMR confirmation represents a tractable near-term research agenda. Specific compound classes worth prioritizing include chlorogenic acid derivatives, luteolin glycosides, and glycosylated phenolic acids, which are known to exhibit bioactivities across anti-inflammatory, antidiabetic, and antioxidant domains [9,11,32]. For the ‘functional soda’ category, the data highlight that distinct ingredient strategies give rise to complementary molecular profiles: vinegar fermentate (Poppi^®^) contributes a rich small-molecule phenolic portfolio, while the fiber-forward botanical blend (OLIPOP^®^) reflects a different but equally deliberate formulation philosophy centered on prebiotic functionality. Together, these profiles suggest that ingredient selection shapes the nature and diversity of detectable bioactive content in category-specific ways. These observations are hypothesis-generating and would require targeted quantification to determine whether the detected compounds are present at functionally relevant concentrations.

### 4.6. Limitations and Future Directions

Several limitations constrain the scope of interpretation. First, the analytical window was restricted to small molecules below 1200 Da, excluding dietary fiber polymers, complex glycoconjugates, and high-molecular-weight polysaccharides that constitute the primary claimed prebiotic activity of OLIPOP^®^, BeePop™ and Poppi^®^. Future orthogonal methods (e.g., glycan arrays, fiber characterization by enzymatic digestion and colorimetric quantitation) are needed for a complete functional picture. Second, signal intensities from untargeted LC–MS/MS are not calibrated against authentic standards at known concentrations and cannot be interpreted as absolute or relative compound concentrations. All inter-beverage comparisons therefore reflect qualitative presence/absence patterns, not concentration differences. Targeted quantification using methods such as isotope-dilution is required to determine whether detected compounds reach physiologically relevant concentrations. Third, the monophasic acetonitrile dilution used for extraction favors compounds with intermediate polarity and may under-represent highly lipophilic or highly polar metabolites, potentially biasing the observed chemical diversity toward mid-polarity compound classes. Fourth, the profiles reported here were obtained from single production lots purchased at one US retail location. While commercial beverages are manufactured under standardized formulations designed for batch consistency, confirmation across multiple production lots and retail sources would further strengthen the representativeness of these findings. Fifth, of the 42 compounds with reported bioactivity, 13 rely on MSI Level 2 annotations, which are putative identifications based on spectral similarity and may include false positives due to isobaric or isomeric co-elution. The tentative detection of L-theanine in all beverages and GABA/citrulline in Coca-Cola^®^ demands confirmatory analysis using isotope-labeled internal standards and authentic reference compounds. Sixth, the bioactive reference database, though curated from peer-reviewed and clinical-trial sources, represents a curated selection of compounds with published biological associations and is inherently subject to selection bias—compounds with more extensive publication histories are more likely to be included. The database should not be interpreted as exhaustive, and the absence of a compound from the database does not imply absence of biological activity.

## 5. Conclusions

This study delivers a systematic, evidence-anchored bioactive profile of commercially available RTD beverages, using dual-mode untargeted LC–MS/MS and MSI-compliant annotation across five products spanning legacy CSDs, prebiotic sodas, and botanical-derived carbonated beverages. Across 478 structurally annotated compounds (MSI Level 1 + 2, from an initial ~3328 detected features), 42 bioactive compounds were matched to an expanded 204-compound bioactive reference database after replicate-presence filtering, spanning amino acids, polyphenols, flavonoids, organic acids, vitamins, and neurotransmitter precursors, including the NAD^+^ precursor trigonelline.

Three themes emerge with clarity from this dataset. First, the number of detected compounds with reported bioactivity appears to reflect ingredient origin more than marketing category: a botanical carbonated beverage (BeePop™) shares a comparable bioactive count with brewed tea (Pure Leaf^®^ Tea), while a legacy CSD (Coca-Cola^®^) and a branded prebiotic soda (OLIPOP^®^) each contribute distinct small-molecule profiles shaped by their respective formulation strategies. Second, polyphenols represent the most prominent compositional differentiator across this beverage set: their presence or absence reflects underlying ingredient choices and distinguishes formulations more clearly than any other detected chemical class. Third, 17 compounds with reported bioactivity—including trigonelline, L-theanine, tryptophan, trehalose, and multiple B vitamins—were detected across all five beverages, though several of these are tentative MSI Level 2 annotations requiring confirmation. Many of these commonly detected compounds are ubiquitous in food matrices and their shared presence may reflect a common aqueous-matrix biochemistry rather than a functionally meaningful feature.

These findings provide a qualitative reference dataset for nutrition research and functional ingredient strategy. The necessary next step is targeted quantitation of the putatively identified bioactives—particularly those annotated at MSI Level 2—to determine whether detected compounds reach biologically relevant concentrations within serving-size volumes, which is the essential link between molecular detection and any potential functional benefit.

## Figures and Tables

**Table 1:** Bioactive compound counts per beverage (MSI Level 1–2 annotations).

Beverage	Total Bioactives Detected	Shared (≥2 Beverages)	Unique to One Beverage
Pure Leaf^®^ Tea (Unsweetened Black Tea)	39	36	2
BeePop™	39	36	1
Poppi^®^ Orange	33	33	0
Coca-Cola^®^	22	22	0
OLIPOP^®^ Cream Soda	28	28	0
Total (union across all beverages)	42	39	3

**Table 2:** Seventeen bioactive compounds robustly detected across all five RTD beverages (≥4/4 replicates, abundance > 10,000). Compounds marked with an asterisk (*) are not declared ingredients in all five beverages and represent unexpected or tentative detections requiring confirmation. Evidence strength classifications (Confirmatory, Suggestive, Preclinical) refer to the level of published evidence for biological activity of each compound in the peer-reviewed literature, not to the confidence of detection in this study. See Section 2.4 for definitions.

Compound	Primary Bioactivity	Evidence Strength
Vitamin C (isoascorbic acid)	Antioxidants; vitamins	Confirmatory
Thiamine (B1)	Vitamins; neuroprotective	Confirmatory
Pyridoxine (B6)	Protective agents; homocysteine-lowering	Confirmatory
Betaine *	Lipotropic; homocysteine-lowering	Confirmatory
Citric acid	Antilithic; antimicrobial; chelating	Confirmatory
Acetylcholine *	Vasodilator; cholinergic agonist	Confirmatory
Tryptophan *	Serotonergic precursor; antidepressive	Confirmatory
D-Ribose/xylose	Cardiac energy substrate	Confirmatory
Galactose/glucose	Energy substrate; diagnostic marker	Confirmatory
Mannitol/sorbitol	Osmotic agents; expectorants	Confirmatory
Sucrose	Dietary carbohydrate	Confirmatory
GABA *	Neurotransmitter; hypoglycemic	Suggestive
L-Theanine *	Nootropic; calming; attention support	Suggestive
Trehalose *	Cytoprotective; neuroprotective	Suggestive
Glucosamine *	Joint/cartilage support; analgesic	Suggestive
Chitin	Biological control; antimicrobial	Suggestive
Trigonelline *	NAD^+^ precursor; muscle function; neuroprotective	Suggestive

**Table 3:** Macronutrient content as reported in product labeling per 12 fluid ounces.

Content	BeePop™ Peach + Orange Blossom Honey	Coca-Cola^®^	OLIPOP^®^ Cream Soda	Poppi^®^ Orange	Pure Leaf^®^ Tea (Unsweetened)
Calories (kcal)	50	140	40	30	0
Total fat (g)	0	0	0	0	0
Sodium (mg)	140	45	30	0	0
Total carbohydrate (g)	13	39	15	9	0
Dietary fiber (g)	2	0	6	3	0
Total sugars (g)	4	39	2	5	0
Added sugars (g)	4	39	2	3	0
Protein (g)	0	0	0	0	0

## Data Availability

The annotated compound dataset (478 MSI Level 1–2 features), replicate-intensity tables, bioactive database mappings, and novel candidate lists are provided as Supplementary Materials. Raw mzML data files will be deposited in the MassIVE public repository (https://massive.ucsd.edu; accessed on 28 May 2026) upon acceptance.

## Supplementary Materials

The following supporting information can be downloaded at https://www.mdpi.com/article/10.3390/metabo16060404/s1, Supplementary Table S1: Full annotated feature table for all five beverages (478 features; MSI Levels 1–2); Supplementary Table S2: Complete bioactive reference database with compound identifiers, biological activity annotations, evidence strength classifications, and beverage presence/absence matrix (42 bioactives); Supplementary Figure S1: Chemical superclass sunburst diagram. Interactive sunburst visualization of ClassyFire chemical taxonomy across all 478 annotated compounds (MSI Level 1 + 2), hierarchically organized from kingdom (outermost ring) through superclass, class, and subclass (inner rings). Sector sizes are proportional to compound counts. The dominant superclass is carboxylic acids and derivatives across all beverages.
