# Supplementary figures and images for: Metabolomic Signatures of Commercial Ready-to-Drink Beverages by Dual-Mode Untargeted LC–MS/MS
Source: Metabolites. 2026 Jun 10;16(6):404. doi: 10.3390/metabo16060404 (PMC13304011; doi:10.3390/metabo16060404)

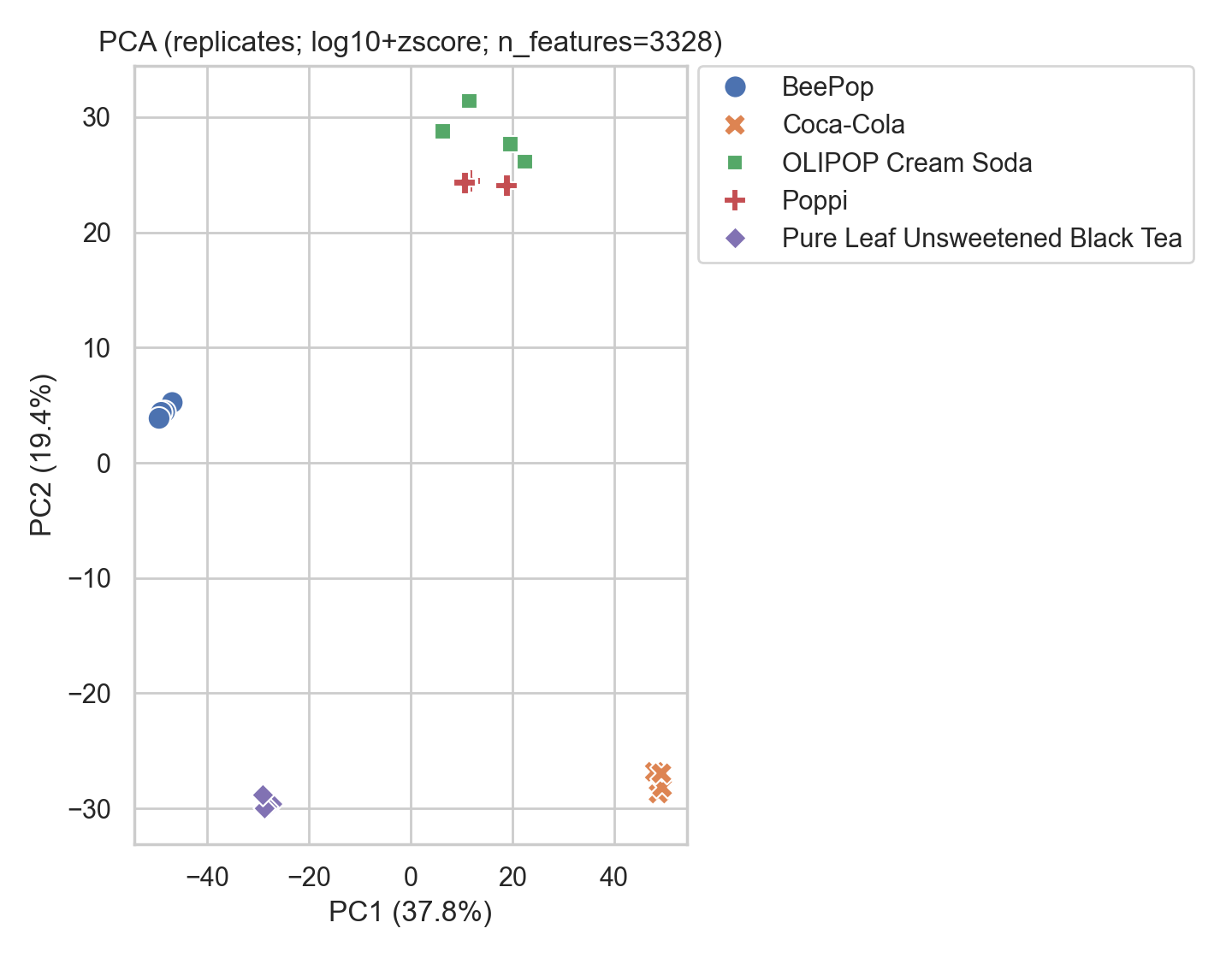

Supplement: Supplementary file 1 [file metabolites-16-00404-s001.zip › supplements/Supplement Figure 1.png]
